# Approach to closure of large mucosal wound after endoscopic resection of duodenal descending lesion: combining endoscopic hand-suturing techniques with metal clip application

**DOI:** 10.1055/a-2724-6696

**Published:** 2025-11-21

**Authors:** Yong Liu, Zeliang Yang, Shun He, Hoi-Loi Ng, Xia Hu, Chengyu Yang, Guiqi Wang

**Affiliations:** 1 12501 Department of Endoscopy, National Cancer Center/National Clinical Research Center for Cancer/Cancer Hospital, Chinese Academy of Medical Sciences and Peking Union Medical College, Beijing, China; 2 Gastroenterology, Mianyang Third People’s Hospital, Sichuan Mental Health Centre, Mianyang, China

Endoscopic submucosal dissection (ESD) is a promising method for achieving complete resection of large superficial duodenal lesions 1 . However, post-ESD wound management is crucial for patient outcomes. If left open, the wound may lead to delayed bleeding, perforation, or infection due to gastric acid, bile, and pancreatic juice. For large wounds, endoscopic metal clip closure or hand-suturing closure alone is often ineffective, while laparoscopic closure increases trauma. This study introduces a novel method combining endoscopic hand suturing with metal clip closure ( Video 1 ).

Demonstration of endoscopic hand-suturing combined with clip closure for the large duodenal descending wound.Video 1

The case involves a high-grade intraepithelial neoplasia in the duodenal descending part, situated just below the papilla and predominantly involving the posterior and both lateral walls ( Fig. 1 **a** ). After obtaining informed consent, ESD was performed ( Fig. 1 **b** ). Post-ESD, the wound measured approximately 8 cm × 6 cm ( Fig. 1 **c** ). The resected specimen measured 8 cm × 5.7 cm ( Fig. 1 **d** ). Initially, endoscopic hand-suturing was used to reduce the wound size to a manageable dimension for metal clip closure ( Fig. 1 **e–g** ). Subsequently, metal clips were applied to reinforce the closure, effectively reducing tension and achieving secure wound closure ( Fig. 1 **h** ). The resected specimen’s pathology confirmed R0 resection. The ESD procedure took 158 minutes, the endoscopic suturing took 58 minutes, and the patient was discharged on postoperative day 14 without complications.

**Fig. 1:**
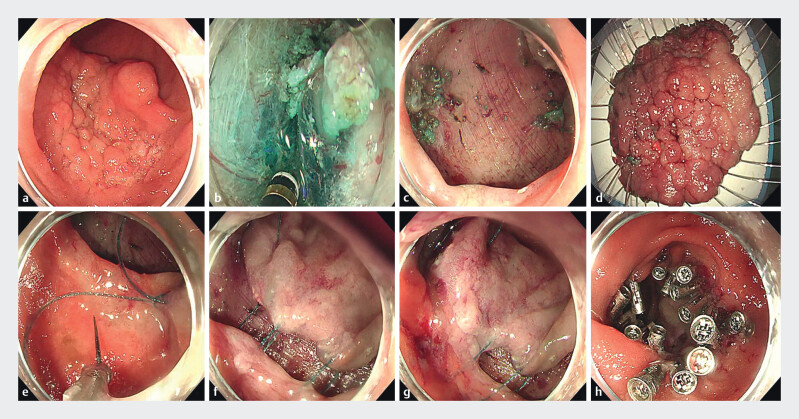
Endoscopic views. **a** An elevated lesion was identified in the descending duodenum. **b** Endoscopic submucosal dissection. **c** The wound after endoscopic submucosal dissection. **d** The resected specimen. **e–f** Endoscopic hand-suturing. **g** The wound after endoscopic hand-suturing. **h** The wound after application of the metal clip closure.

This study is the first to report the use of endoscopic hand-suturing combined with metal clip closure for a large duodenal descending part wound post-ESD. This approach offers a feasible alternative to laparoscopic closure, reducing surgical time and trauma while avoiding excessive.

Endoscopy_UCTN_Code_TTT_1AO_2AO

## Notes

Endoscopy E-Videos https://eref.thieme.de/e-videos
*E-Videos* is an open access online section of the journal *Endoscopy* , reporting on interesting cases and new techniques in gastroenterological endoscopy. All papers include a high-quality video and are published with a Creative Commons CC-BY license. Endoscopy E-Videos qualify for HINARI discounts and waivers and eligibility is automatically checked during the submission process. We grant 100% waivers to articles whose corresponding authors are based in Group A countries and 50% waivers to those who are based in Group B countries as classified by Research4Life (see: https://www.research4life.org/access/eligibility/ ). This section has its own submission website at https://mc.manuscriptcentral.com/e-videos .
